# 

**Published:** 1892-06

**Authors:** 


ESTABLISHED 1855. SUBSCRIPTION, $2.00.

### ATLANTA MEDICAL AND SURGICAL JOURNAL.

OLD SERIES, VOL. XXVI. NEW SERIES, VOL. IX. | ATLANTA, GA., JUNE, 1892. No. 4.

LUTHER B. GRANDY, M. D., MANAGING EDITOR.

M. B. HUTCHINS, M. D., BUSINESS MANAGER.

